# Molecular Mechanisms of Metformin for Diabetes and Cancer Treatment

**DOI:** 10.3389/fphys.2018.01039

**Published:** 2018-07-31

**Authors:** Min Li, Xiaoying Li, Huijie Zhang, Yan Lu

**Affiliations:** ^1^Department of Endocrinology and Metabolism, Zhongshan Hospital, Fudan University, Shanghai, China; ^2^Department of Endocrinology and Metabolism, Nanfang Hospital, Southern Medical University, Guangzhou, China

**Keywords:** metformin, type 2 diabetes, gluconeogenesis, hepatic glucose production, cancer, cell proliferation, AMPK, mTOR

## Abstract

Metformin has been the first-line drug treatment for hyperglycemia and insulin resistance for over 50 years. However, the molecular basis of its therapeutic role remained incompletely understood. Recent advances demonstrate that metformin could exert its glucose-lowering effect by multiple mechanisms, including activation of 5′-AMP-activated protein kinase, decreasing production of cyclic AMP, suppressing mitochondrial complex I of the electron transport chain, targeting glycerophosphate dehydrogenase, and altering the gut microbiome. In addition, epidemiological and clinical observation studies suggest that metformin reduced cancer risk in patients with type 2 diabetes and improved survival outcome of human cancers. Experimental studies have shown that this drug can inhibit cancer cell viability, growth, and proliferation through inhibiting mTORC1 signaling and mitochondrial complex I, suggesting that it may be a promising drug candidate for malignancy. Here, we summarize recent progress in studies of metformin in type 2 diabetes and tumorigenesis, which provides novel insight on the therapeutic development of human diseases.

## Introduction

Metformin, a derivative of guanidine, has been used in the treatment of hyperglycemia and type 2 diabetes mellitus (T2DM) for over 50 years (American Diabetes Association, 2014). Metformin, phenformin, and buformin are derivatives of guanidine, which was extracted from the plant isoamylene in the 1920s (Bailey and Day, 1989). Phenformin and buformin have been withdrawn in the early 1970s because of their higher risk of cardiac mortality and lactic acidosis (Luft et al., 1978), whereas the use of metformin has been expanded from T2DM to polycystic ovary disease, diabetic nephropathy, gestational diabetes, T2DM-associated cardiovascular complications (Viollet et al., 2012), due to its superior safety profile.

Metformin specifically suppresses hepatic gluconeogenesis without increasing the burden of pancreatic β cells to enhance insulin secretion or promoting adipocyte differentiation to induce weight gain (Inzucchi et al., 1998). However, the exact molecular mechanisms of its glucose-lowering effects remain unclear. Besides, metformin has gained attention for its pleiotropic effects. Importantly, accumulation of numerous epidemiological studies indicates the preventive and therapeutic effects of metformin on many types of human cancers (Morales and Morris, 2015). Herein, we summarized the action and molecular mechanisms through which metformin inhibits hepatic gluconeogenesis and tumorigenesis, which may help to suggest directions for future investigation.

## Molecular Mechanisms of Anti-Diabetic Effects

**FIGURE 1:**
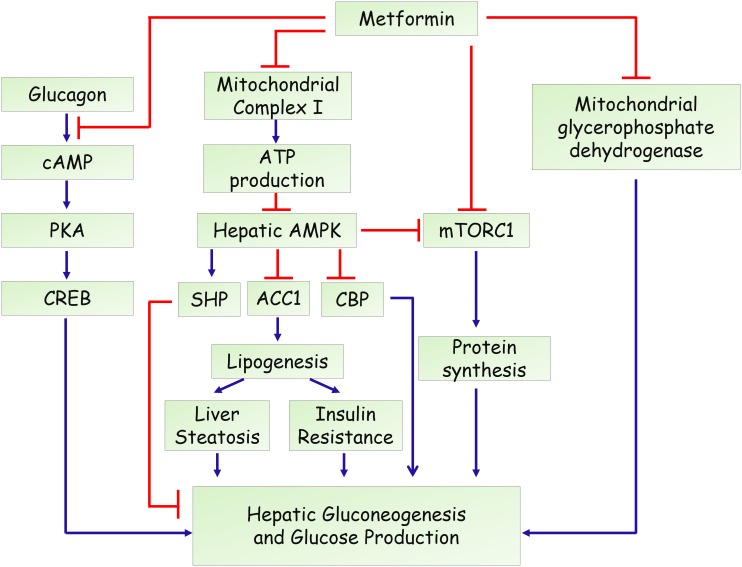
Proposed actions of metformin in the hepatic gluconeogenesis.

In the past decade, several mechanisms have been identified for the action of metformin in hepatic gluconeogenesis and glucose production (**Figure 1**). An important breakthrough was that metformin could activate adenosine 5′-monophosphate (AMP)-activated protein kinase (AMPK) (Zhou et al., 2001), a master regulator of various metabolic pathways (Hardie et al., 2012; Lin and Hardie, 2018), by increasing its phosphorylation at Thr-172. Through screening of a compound library containing more than 10,000 molecules, compound C was discovered as an AMPK inhibitor and attenuated metformin’s effect in hepatocytes (Zhou et al., 2001), indicating that activation of AMPK is essential for its inhibitory effects on glucose production in hepatocytes. A subsequent study by Shaw et al. (2005) reported that deletion of liver kinase B1 (LKB1), the upstream kinase that phosphorylates and activates AMPK, led to a nearly complete loss of AMPK activity in the liver of adult mice. Loss of LKB1 blocked the therapeutic effects of metformin, suggesting that metformin treatment of mice increased AMPK activity in the liver and lowered blood glucose levels in an LKB1-dependent manner (Shaw et al., 2005). Besides, some studies demonstrated that metformin treatment increases cellular levels of AMP through suppressing complex I of the mitochondrial electron transport chain. This inhibition resulted in a reduced cellular ATP concentrations and an elevated AMP levels (Batandier et al., 2006). Moreover, low concentrations of metformin was shown to promote the formation of the AMPK αβγ complex through augmenting phosphorylation by LKB1 and antagonizing dephosphorylation by PP2C, leading to the phosphorylation of the AMPK α catalytic subunit at Thr-172 (Meng et al., 2015). Moreover, one recent study revealed that metformin activates AMPK through the lysosomal pathway, consisting of AXIN/LKB1-v-ATPase-Ragulator pathway (Zhang et al., 2016). Therefore, the mechanisms for metformin to activate AMPK remain obscure and controversial. On the other hand, the molecular mechanism underlying the AMPK-mediated inhibition of gluconeogenesis remained elusive. A study from Choi et al. showed that metformin suppresses hepatic glucose production and expression of gluconeogenic genes (PEPCK and G6Pase) through AMPK-dependent upregulation of small heterodimer partner (SHP), a transcriptional co-repressor (Kim et al., 2008). SHP could interact with and repress the transcriptional activity of hepatocyte nuclear factor 4α (HNF4α), forkhead box protein O1 (FoxO1), and forkhead box protein A2 (FoxA2), which are critical in the transcriptional regulation of gluconeogenic genes (Rines et al., 2016). Another study demonstrated that metformin inhibits hepatic gluconeogenesis through phosphorylation of CREB binding protein (CBP) at serine 436 via AMPK-PKCι/λ, leading to the dissociation of the CREB-CBP-CRTC2 transcription complex and down-regulation of gluconeogenic genes (He et al., 2009). In addition, AMPK could phosphorylate acetyl-CoA carboxylase 1 (ACC1) and ACC2 to inhibit the conversion of acetyl-CoA to malonyl-CoA. As a result, AMPK activation by metformin results in reduced liver lipogenesis and hepatosteatosis, contributing to improved insulin resistance and hyperglycemia (Fullerton et al., 2013; Ford et al., 2015; Boudaba et al., 2018). Together, all these studies highlight the importance of AMPK signaling in the anti-diabetic action of metformin (Rena et al., 2017).

The controversy arised in 2010 when Foretz et al. (2010) showed that metformin could inhibit hepatic gluconeogenesis in mice lack of either AMPKα1α2 catalytic isoforms or LKB1. They found that the hypoglycemic effect of metformin was unaltered in liver AMPK deficient mice, compared with wild-type mice (Foretz et al., 2010). Consistently, reduced expression of gluconeogenic genes by metformin was also comparable in the wild-type, AMPKα1α2 deficient, and LKB1 deficient hepatocytes, further confirming that neither AMPK nor LKB1 are required for metformin-mediated suppression of hepatic gluconeogenesis (Foretz et al., 2010). However, a recent study questioned the high or supra-pharmacological concentrations of metformin in Foretz’s study and found that low concentrations of metformin (60–80 μM in the portal vein of animals) suppress glucose production via an AMPK dependent mechanism (Cao et al., 2014). In agreement, Howell et al. (2017) showed that low doses of metformin inhibit the mammalian target of rapamycin complex I (mTORC1) through AMPK and higher doses act through alternative mechanisms. Therefore, it is speculated that the mechanisms of metformin might be associated with its concentrations (doses) (He and Wondisford, 2015).

On the other hand, Cokorinos et al. (2017) demonstrated that direct pharmacological activation of AMPK by small-molecule AMPK activators in liver is not sufficient for acute glucose lowering in obese mice. Thus, an important question raised by these work is that how metformin could lower blood glucose or hepatic glucose production in the absence of AMPK. Foretz et al. (2010) proposed that the action of metformin is attribute to decreased cellular ATP concentrations and increased AMP/ATP ratio. Besides, Miller et al. (2013) reported that metformin could antagonize the role of glucagon, to reduce blood glucose levels. They found that treatment of metformin could increase levels of AMP and related nucleotides to suppress adenylate cyclase and protein kinase A activity, abolish CREB phosphorylation, and block glucagon-stimulated glucose output in hepatocytes (Miller et al., 2013). Madiraju et al. (2014) demonstrated that metformin could suppress the mitochondrial glycerophosphate dehydrogenase (mGPD), leading to reduced conversion of lactate and glycerol to glucose. Furthermore, recent studies implicates that the gastrointestinal tract may be involved in the antidiabetic effect of metformin (Bailey et al., 2008). It has been shown that preabsorptive metformin could activate duodenal mucosal AMPK to inhibit hepatic gluconeogenesis and improve hyperglycemia in high-fat-diet-induced obese rodents (Duca et al., 2015; Jensen et al., 2016). Duodenal infection of an adenovirus containing the dominant negative AMPK largely attenuated the glucose lowering effect of intraduodenal metformin (Duca et al., 2015). Moreover, metformin upregulates the expression levels of sodium glucose cotransporter-1 (SGLT1) in upper small intestine, partly by increasing the abundance of Lactobacillus (Bauer et al., 2018). In addition, two studies using T2DM patients further implicates the gut microbiota as an important site of metformin action (Forslund et al., 2015; Wu et al., 2017). Importantly, a randomized, placebo-controlled, double-blind study in newly diagnosed T2DM subjects showed that metformin had rapid effects on the composition and function of the gut microbiota (Wu et al., 2017). Animal experiments further confirmed that transfer of the metformin-altered microbiota to germ-free mice could improve glucose metabolism (Wu et al., 2017), suggesting that altered gut microbiota contributes to the therapeutic effects of the drug.

## Molecular Mechanisms of Antineoplastic Effects

Dilman et al. (1978) found that daily oral administration of phenformin suppressed dimethylbenzathracene-induced mammary tumor development in rats. They further reported that breast cancer patients taking phenformin had an improvement in metabolic parameters and immunologic status (Dilman et al., 1982). In recent years, lots of epidemiological studies looked into the preventive and therapeutic actions of metformin on many types of human cancers. The first report was a case–control study showing a decreased risk of developing cancer in T2DM patients taking metformin (Evans et al., 2005), which was further confirmed by subsequent meta-analysis using 18 observational studies in liver, colon, and pancreatic cancers (Franciosi et al., 2013). In addition to its preventive action, the beneficial effect of metformin on improvement of overall survival outcomes or reduction in mortality was also observed in liver, pancreatic, colorectal, and breast cancer (Zhang et al., 2013; Morales and Morris, 2015), suggesting that it can also serve as a potential anti-tumor agent (Jiralerspong et al., 2009). For instance, a study involving 1,013 breast cancer patients showed that the HER-2 positive rate was lower in the metformin-treated group than in the nonmetformin-treated group (Hou et al., 2013). Besides, metformin-treated group was associated with better clinical outcomes and lower mortality risk (Hou et al., 2013).

Although the use of metformin is still limited to T2DM, insulin resistance and hyperglycemia, its effect in non-diabetic cancer patients was also observed. It was reported that metformin inhibited colonic epithelial proliferation and reduced rectal aberrant crypt foci in non-diabetic patients with colorectal cancer (Hosono et al., 2010). Besides, Hadad et al. (2011, 2015) performed a pre-operative trial, which provides support for anti-proliferative effects of metformin in non-diabetic breast cancer humans. In addition, recent *in vitro* and *in vivo* studies indicate that metformin can enhance the effects of other anti-cancer drug, such as cisplatin, vincristine, 5-fluorouracil, and doxorubicin (Iliopoulos et al., 2011; Miranda et al., 2014; Yi et al., 2017; Candido et al., 2018), suggesting metformin can act as part of combinatorial therapy to decrease the chemotherapy dose in cancer patients.

**FIGURE 2:**
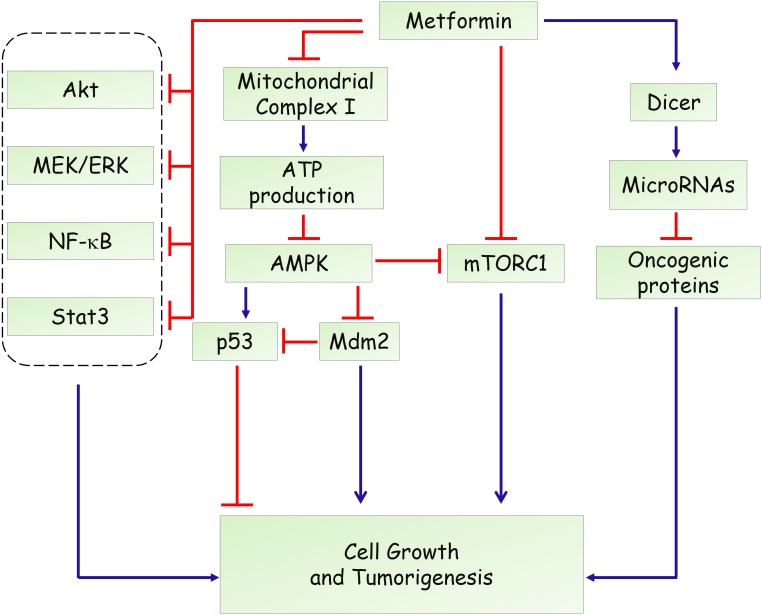
Potential mechanisms of metformin in anti-tumorigenesis.

Hyperinsulinemia represents a risk factor for several types of human malignancy and induces adverse prognosis (Pollak, 2012; Garg et al., 2014). Therefore, systemic effects related to reduced blood glucose levels, improved insulin resistance and decreased pro-inflammatory cytokines, are involved in the complexity of the roles of metformin on tumorigenesis (Pernicova and Korbonits, 2014). Besides, a direct action of metformin in cancer cells needs more attention. Likewise, the anti-diabetic actions, initial studies showed that LKB1-dependent and AMPK-dependent growth inhibitor was responsible for the antineoplastic effect of metformin (**Figure 2**) (Zakikhani et al., 2006; Dowling et al., 2007). Knockdown of AMPK α1 subunit by small interfering RNA rescued breast and ovarian cancer cells from the inhibitory effect of metformin (Zakikhani et al., 2006). AMPK activation induces phosphorylation of p53 on Ser15, and this phosphorylation is required to initiate AMPK-dependent cell-cycle arrest (Jones et al., 2005). Activation of AMPK by metformin also promotes phosphorylation of human MDMX on Ser342, which inhibits p53 ubiquitylation and stabilizes p53 (He et al., 2014). However, a subsequent study found that the antiproliferative role of metformin is not mediated by AMPK in prostate cancer cells and proposed that inhibition of mTOR represents an alternative pathway for metformin action (Ben Sahra et al., 2011). mTOR is a catalytic subunit of two multiprotein complexes, mTORC1 and mTORC2, which integrate both intracellular and extracellular stimuli and act as a key regulator of cell growth (Laplante and Sabatini, 2012; Saxton and Sabatini, 2017). mTOR inhibition could disturb protein synthesis, and thereby suppress tumor cell proliferation. Metformin was shown to suppress the activation of mTOR through AMPK-dependent and -independent mechanisms. AMPK-dependent suppression of mTORC1 activity is attributed to activation of tuberous sclerosis complex 1 (TSC1) and TSC2, which form an mTOR-inhibiting complex (Inoki et al., 2003). Moreover, AMPK could directly phosphorylate Raptor, the mTOR binding partner protein, which is required for the inhibition of mTORC1 induced by energy stress (Gwinn et al., 2008). In addition, Kalender et al. (2010) reported that metformin can inhibit mTORC1 signaling through Ras-related GTPase, independent of AMPK and TSC1/2. In addition to AMPK and mTOR, metformin has been shown to affect other oncogenic signaling pathways. Li and colleagues reported that metformin suppresses the proliferation and growth of osteosarcoma and renal cell carcinoma cells by suppressing Akt phosphorylation, which was associated with increased phosphatase and tensin (PTEN) expression (Kalogirou et al., 2016; Li et al., 2018). Besides, metformin could activate the MEK/ERK signaling pathway to promote leukemia cell differentiation and apoptosis (Huai et al., 2012). Moreover, metformin inhibits activation of NF-κB and Stat3 signalings in cancer stem cells, resulting in a reduced inflammatory response and attenuated tumor growth (Hirsch et al., 2013).

Furthermore, modulation of microRNA expression has been proposed to underlie the anticancer actions of metformin. It has been reported that metformin treatment could induce the expression of DICER, an enzyme that is crucial in regulating microRNA biogenesis (Blandino et al., 2012). Downregulation of DICER has been shown to represent an intrinsically oncogenic event and predicts poor survival of several types of cancers (Karube et al., 2005; Merritt et al., 2008; Martello et al., 2010). Forced overexpression of DICER recapitulated the antineoplastic role of metformin *in vitro* and *in vivo*. Besides, the effects of metformin are substantially impaired in DICER-deficient tumor cells (Blandino et al., 2012), suggesting that upregulation of DICER is required for its actions. As a result, metformin treatment affected expression levels of many microRNAs such as miR-21, miR-26a, miR-33a, miR-140-5p, miR-142-3p, miR-181a, miR-192, miR-193b, R-20mi0, miR-205, miR-222, let-7a, and let-7c, which further modulates several target genes in metabolic or preoncogenic pathways (Pulito et al., 2014; Zhou et al., 2015).

Moreover, a recent study showed that the environment drastically altered sensitivity to metformin (Gui et al., 2016). They demonstrated that complex I supports proliferation by regenerating nicotinamide adenine dinucleotide (NAD) and metformin’s anti-proliferative effect is due to loss of NAD/NADH homeostasis and inhibition of aspartate biosynthesis (Gui et al., 2016). In agreement, through an integrative metabolomics analysis of metformin action in ovarian cancer, Liu et al. (2016) showed that metformin could target central carbon metabolism, suggesting mitochondrial requirements for the effects of metformin on cancer cells. In addition, through genetic screening in *C. elegans*, Wu et al. (2016) identified two metformin response elements: the nuclear pore complex (NPC) and acylCoA dehydrogenase family member-10 (ACAD10). They demonstrated that metformin inhibited cell growth by inhibiting mitochondrial respiratory capacity, which restrains transit of the RagA-RagC GTPase heterodimer through the NPC (Wu et al., 2016). Together, these findings not only provide precise indications of metformin in cancer but also uncover new insights into mitochondrial metabolism.

Overall, the anti-proliferative effects of metformin share common mechanisms with its anti-diabetic action, including activation of AMPK signaling, inhibition of mTOR signaling, targeting mitochondria complex I (**Figures 1, 2**). Although the detailed reason remains poorly understood, we speculate that a unified mechanism might exist in metformin-treated normal cells and cancer cells, such as the alteration of cellular energy state. In addition, several studies also demonstrated that use of metformin is not associated with reduced incidence or improved outcome in certain types of human cancers (Mamtani et al., 2014; Suissa and Azoulay, 2014; Kordes et al., 2015). Therefore, the therapeutic effect of metformin might be cell- or tissue-specific, which needs to be determined in future studies.

## Conclusion

Metformin has been widely used in the treatment of T2DM and related metabolic diseases. However, as reviewed here, both AMPK-dependent and AMPK-independent pathways have been proposed to explain the glucose-lowering and anti-tumor effect of metformin (**Figures 1, 2**). Besides, although the liver is considered as the primary site of metformin pharmacodynamics, the gut is also recognized an important site for its anti-diabetic and anti-tumor effects (Duca et al., 2015; Paleari et al., 2018). In addition, recent studies demonstrated that metformin might affect metabolite profiles in patients with type 2 diabetes or tumor cells (Zhang et al., 2014; He et al., 2015; Xu et al., 2015). All these knowledge, we hope, will help to fully understand the mechanistic action of metformin, which may propel the development of novel potential therapeutic targets in treating human diseases.

## Author Contributions

ML, XL, HZ, and YL drafted the manuscript. YL and HZ handled funding and supervision. All authors reviewed the manuscript.

## Conflict of Interest Statement

The authors declare that the research was conducted in the absence of any commercial or financial relationships that could be construed as a potential conflict of interest.
